# Identification of genetic susceptibility for Chinese migraine with depression using machine learning

**DOI:** 10.3389/fneur.2024.1418529

**Published:** 2024-07-31

**Authors:** Xingkai An, Shanshan Zhao, Jie Fang, Qingfang Li, Cen Yue, Chuya Jing, Yidan Zhang, Jiawei Zhang, Jie Zhou, Caihong Chen, Hongli Qu, Qilin Ma, Qing Lin

**Affiliations:** ^1^Department of Neurology and Department of Neuroscience, The First Affiliated Hospital of Xiamen University, School of Medicine, Xiamen University, Xiamen, China; ^2^Fujian Key Laboratory of Brain Tumors Diagnosis and Precision Treatment, Xiamen, China; ^3^Xiamen Key Laboratory of Brain Center, Xiamen, China; ^4^Xiamen Medical Quality Control Center for Neurology, Xiamen, China; ^5^Fujian Provincial Clinical Research Center for Brain Diseases, Fuzhou, China; ^6^Xiamen Clinical Research Center for Neurological Diseases, Xiamen, China; ^7^Department of Pediatrics, The First Affiliated Hospital of Xiamen University, Xiamen, China

**Keywords:** migraine, depression, genetic susceptibility, polymorphism, machine learning

## Abstract

**Background:**

Migraine is a common primary headache that has a significant impact on patients’ quality of life. The co-occurrence of migraine and depression is frequent, resulting in more complex symptoms and a poorer prognosis. The evidence suggests that depression and migraine comorbidity share a polygenic genetic background.

**Objective:**

The aim of this study is to identify related genetic variants that contribute to genetic susceptibility to migraine with and without depression in a Chinese cohort.

**Methods:**

In this case-control study, 263 individuals with migraines and 223 race-matched controls were included. Eight genetic polymorphism loci selected from the GWAS were genotyped using Sequenom’s MALDI-TOF iPLEX platform.

**Results:**

In univariate analysis, *ANKDD1B* rs904743 showed significant differences in genotype and allele distribution between migraineurs and controls. Furthermore, a machine learning approach was used to perform multivariate analysis. The results of the Random Forest algorithm indicated that *ANKDD1B* rs904743 was a significant risk factor for migraine susceptibility in China. Additionally, subgroup analysis by the Boruta algorithm showed a significant association between this SNP and migraine comorbid depression. Migraineurs with depression have been observed to have worse scores on the Beck Anxiety Inventory (BAI) and the Migraine Disability Assessment Scale (MIDAS).

**Conclusion:**

The study indicates that there is an association between *ANKDD1B* rs904743 and susceptibility to migraine with and without depression in Chinese patients.

## Introduction

Migraine is a chronic neurological disability with a range of symptoms such as nausea, vomiting, photophobia, phonophobia and sensorimotor disturbances, with each attack lasting 4–72 h (1). It affects more than 1 billion people worldwide and causes a heavy family and socio-economic burden (2, 3). The 1-year prevalence of migraine in China is 9.3%, resulting in 5.5 million life years of disability, and a majority of young women (4, 5). The etiology of migraine remains uncertain, and there is strong evidence of a genetic predisposition (6).

Recent clinical research has found that migraine is associated with an increased risk of several other disorders, such as stroke, asthma, depression, anxiety, sleep disorders, restless leg syndrome and medication overuse headache (7, 8). A cross-sectional study of comorbidities in out-patient headache patients at the Headache Center of the First Affiliated Hospital of Xiamen University showed that migraine patients with depression had poor sleep quality. Depressive disorders are equally disabling as migraine, and there is a bidirectional correlation between the two as risk factors for each other. A 2019 meta-analysis, which included 4 cohort studies and 12 cross-sectional studies, found that migraine increases the risk of depressive disorders by 2-fold (9). In another large-sample study from Taiwan region, migraineurs and their non-migraine siblings were more likely to be depressed, and those with depression and their unaffected siblings had a significantly increased risk of developing migraine (10). The cause of the co-occurrence of migraine and depressive disorders is not yet fully understood. The increased risk of interaction between these two diseases may be related to common genetic, environmental and pathological mechanisms (11).

Over the years, several genome-wide association studies (GWAS) have been conducted for migraine and depression. In 2018, Yang et al. conducted a study using single nucleotide polymorphisms (SNPs) and gene-based analyses of GWAS genotypic data, which included 30,465 cases of migraine, and 75,607 cases of major depressive disorder (MDD), and found that the two disorders have significant genetic overlap and significant cross-disease genetic correlations between the two disorders, and three SNPs (rs146377178, rs672931, and rs11858956) were found to have novel genome-wide significant associations with migraine and MDD. Furthermore, two genes, *ANKDD1B* and *KCNK5*, produced Fisher’s combined gene-based *p*-values that surpassed the genome-wide significance threshold (12). Research conducted by the Brainstorm Consortium confirms that migraine, unlike other neurological disorders, shares more common genetic structures with mental disorders (13). Using the largest sample to date and novel statistical tools (including 59,674 migraine patients and 316,078 controls), Shahram et al. aimed to determine the extent to which the polygenic structure of migraine overlaps with depression and other psychiatric disorders, and identified 14 genetic loci that are commonly associated with migraine and depression (14).

Numerous GWAS studies have produced varying results, possibly due to differences in populations, research methods, and statistical tools. This reflects the complexity of exploring genetic susceptibility to disease. Our previous studies have confirmed the existence of a genetic predisposition in the Chinese migraine population that is partially identical to that in European and American populations (15–17). Given the recent findings and the paucity of relevant studies in China, we investigated the relevance of several loci of interest in the GWAS study to comorbid depression in migraineurs in southern Fujian province of China.

## Materials and methods

### Subjects

A total of 266 migraine patients were recruited from March 2021 to June 2023 at the Headache Centre of the Department of Neurology, the First Affiliated Hospital of Xiamen University. The third edition of the International Classification of Headache Disorders (ICHD-3) was used for diagnostic criteria, and all patients underwent a detailed clinical assessment, including age, gender, disease duration, and major migraine-related scales such as the Migraine Disability Assessment (MIDAS), Visual Analogue Scale (VAS), Beck Depression Inventory (BDI), and Beck Anxiety Inventory (BAI). Subjects with comorbidities such as tumors, other chronic medical conditions, and psychiatric disorders other than depressive disorders were excluded. Two hundred thirty-three controls were nurses and healthy volunteers from our hospital who had a routine medical check-up for non-migraine headaches and were able to complete the BDI assessment to exclude depressive disorders. The two groups were matched for age and gender and came from the same geographical area. Genomic DNA was extracted from peripheral blood lymphocytes using the TIANamp Blood DNA Kit (Tiangen Biotech, Beijing, China) and stored at −80°C for genotyping. Written informed consent was obtained from all participants and the study was approved by the hospital ethics committee (XMYY-2021KYSB009).

### Selected SNPs and genotyping

The tagged SNPs were derived from the GWAS conducted by Yang et al. in 2018 and included rs34358 (*ANKDD1B*), rs904743 (*ANKDD1B*), rs9394578 (*KCNK5*) and rs2815095 (*KCNK5*) (12). Additionally, the most strongly associated loci with migraine and depression from the GWAS carried out by Shahram et al. in 2022 were taken into account in this study, including rs1217091, rs7592120, rs11210247, rs71327107 (14). All of these SNPs were selected because their minor allele frequency (MAF) was greater than 0.15. SNP genotyping was performed on all subjects using Sequenom iPLEX Assay technology (Sequenom, San Diego, CA, United States).

### Statistical analysis

Statistical analysis was conducted using SPSS 23.0 and R4.3.2^1^ software. The minimum sample size required for this study was calculated using the “pmsampsize” R package. Univariate analysis was conducted using the t-test and Chi-square test to compare the data of two groups. Multivariate analysis was performed by constructing a random forest model using the “randomForest” package. The data set was bootstrapped multiple times to form a training set. The importance of sample features was evaluated using Mean Decrease Accuracy and Gini index as observation indices. The “Boruta” package was used to extract and sort the important features of the random forest model. The Boruta algorithm underwent multiple iterations to evaluate the scores of all features and determine whether their importance exceeded that of the shadow feature. In the outcome analysis, the green variables were found to be the most significant, while the yellow variables were deemed controversial and the red variables were rejected. The study analyzed the important variables using a generalized linear model (GLM), and the statistically significant risk factors were then screened and quantified using multivariate analysis.

The public statistical web tool https://wpcalc.com/en/equilibrium-hardy-weinberg/ was used to verify Hardy–Weinberg equilibrium for all polymorphisms in the control group. To account for errors in multiple comparisons, a Bonferroni correction was applied.

## Results

### Sample size estimation

Using the “pmsampsize” R package, the parameters were set as follows: eight candidate parameters, assuming a projected migraine prevalence of 0.15 and a *C*-statistic of 0.90, the minimum required sample size was calculated to be 235.

### Baseline patient information

A total of 266 migraine cases were included, of which 236 were female (88.7%) and 30 were male (11.3%). The mean age of migraine was 34.0 ± 9.3 years, including 36 migraine with aura (13.5%) and 230 migraine without aura (86.5%). The 223 controls included 32 men and 191 women with a mean age of 33.1 ± 8.5 years. There were no significant differences in gender and age between the two groups (*p* = 0.309 and *p* = 0.245, respectively). According to the Beck Depression Inventory, 108 people with depression lasting more than 2 weeks were considered to have a depressive disorder, of which 43 were mild, 39 moderate and 26 severe (Supplementary Table S1).

### Univariate analysis

All SNPs were well detected, and the genotypes of all SNPs in the control group were consistent with Hardy–Weinberg balance. The genotype and allele distribution of *ANKDD1B* rs904743 differed significantly between migraine patients and controls (Supplementary Table S2). Among migraine patients, 81.8% carried the rs904743 risk A allele, which was significantly higher than the control group (OR = 1.415, 95% CI: 1.039–1.929, *p* = 0.027). In addition, other polymorphisms did not differ significantly in genotype and allele frequency distribution between migraine cases and controls.

### Machine learning analysis

**Figure 1:**
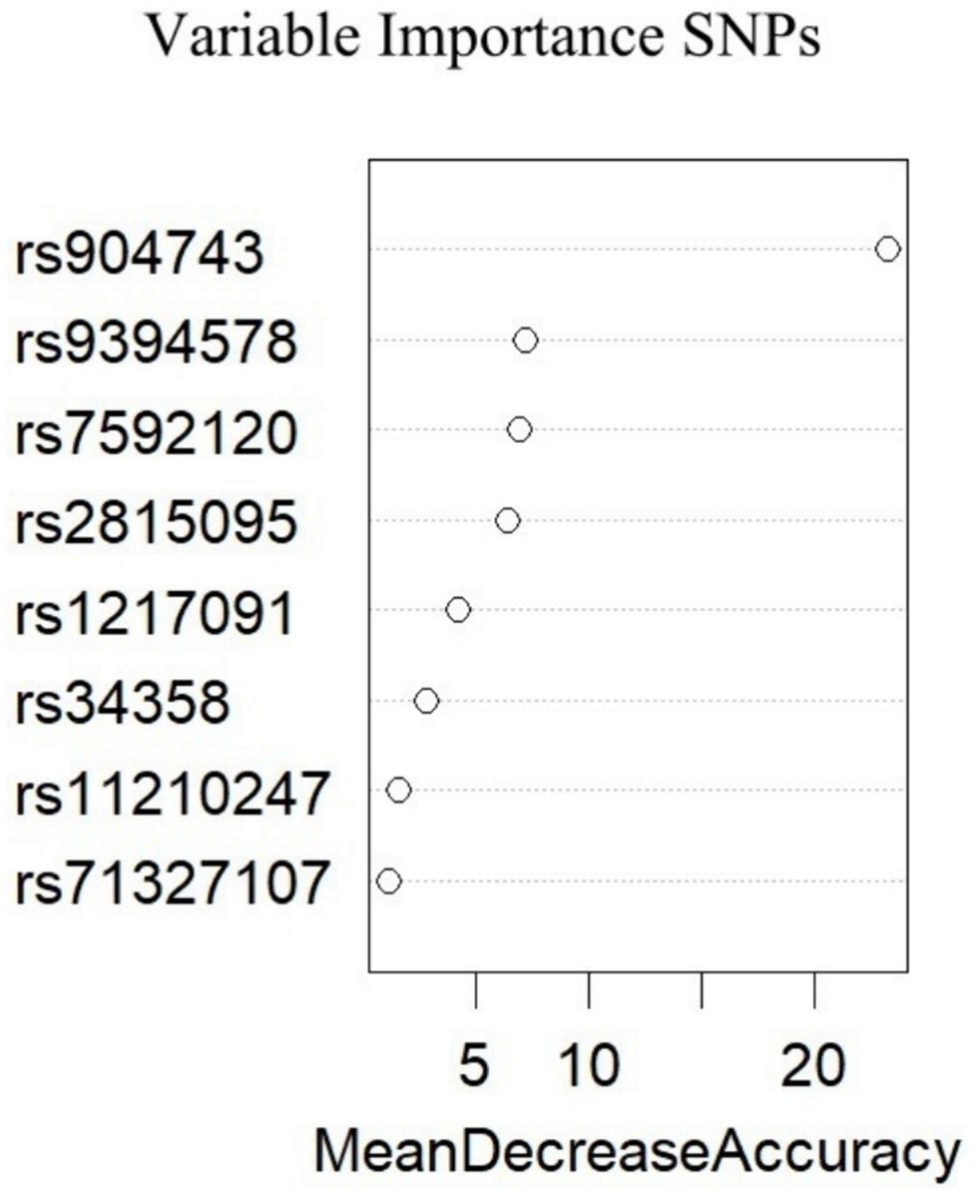
Importance of variables for all migraine derived from the Random Forest. A greater value of mean decrease accuracy indicates a greater importance of the variables.

To investigate whether the selected SNPs were risk factors for migraine in the multivariate analysis, machine learning based on big data analysis was used in this study. The presence of migraine was used as the dependent variable, and the selected SNPs were included in the random forest model as independent variables. The collected dataset was divided into two datasets with a ratio of 0.8:0.2, and the number of random seeds was set. A random forest model was used for training and the model ranked the importance of risk factors. The results showed that the top 4 risk factors were rs904743, rs9394578, rs7592120 and rs2815095, as shown in Figure 1. The results of the generalized linear model analysis indicated that rs904743 was a risk factor for the migraine group (OR = 1.36, 95% CI: 1.01–1.82, *p* = 0.040), whereas rs9394578, rs7592120, and rs2815095 were not statistically significant factors (Table 1).

**Table 1:** Selected susceptibility SNPs for migraine in GLM Logistic analysis.

SNP	Gene	Risk allele	All migraine^c^			Migraine with depression^d^		
			OR^a^	95% CI	*p*	OR^a^	95% CI	*p*_corr_ ^b^
rs904743	*ANKDD1B*	A	1.36	1.01–1.82	**0.040**	1.64	1.08–2.50	**0.021**
rs9394578	*KCNK5*	A	0.54	0.22–1.33	0.179	0.25	0.06–1.01	0.051
rs2815095	*KCNK5*	T	1.61	0.65–3.97	0.301	3.61	0.86–15.22	0.080
rs7592120	—	T	1.09	0.76–1.56	0.643	1.13	0.71–1.80	0.609

SNP, single nucleotide polymorphism; OR, odds ratio; CI, confidence interval.

a OR was the adjusted OR after multiple regression.

b After Bonferroni correction, significance was taken at *p*_corr_ ≤ 0.05/2 in two subgroups; bold value denote significance.

c No. of observations = 489, AIC value = 677.399.

d No. of observations = 331, AIC value = 416.696.

**Figure 2:**
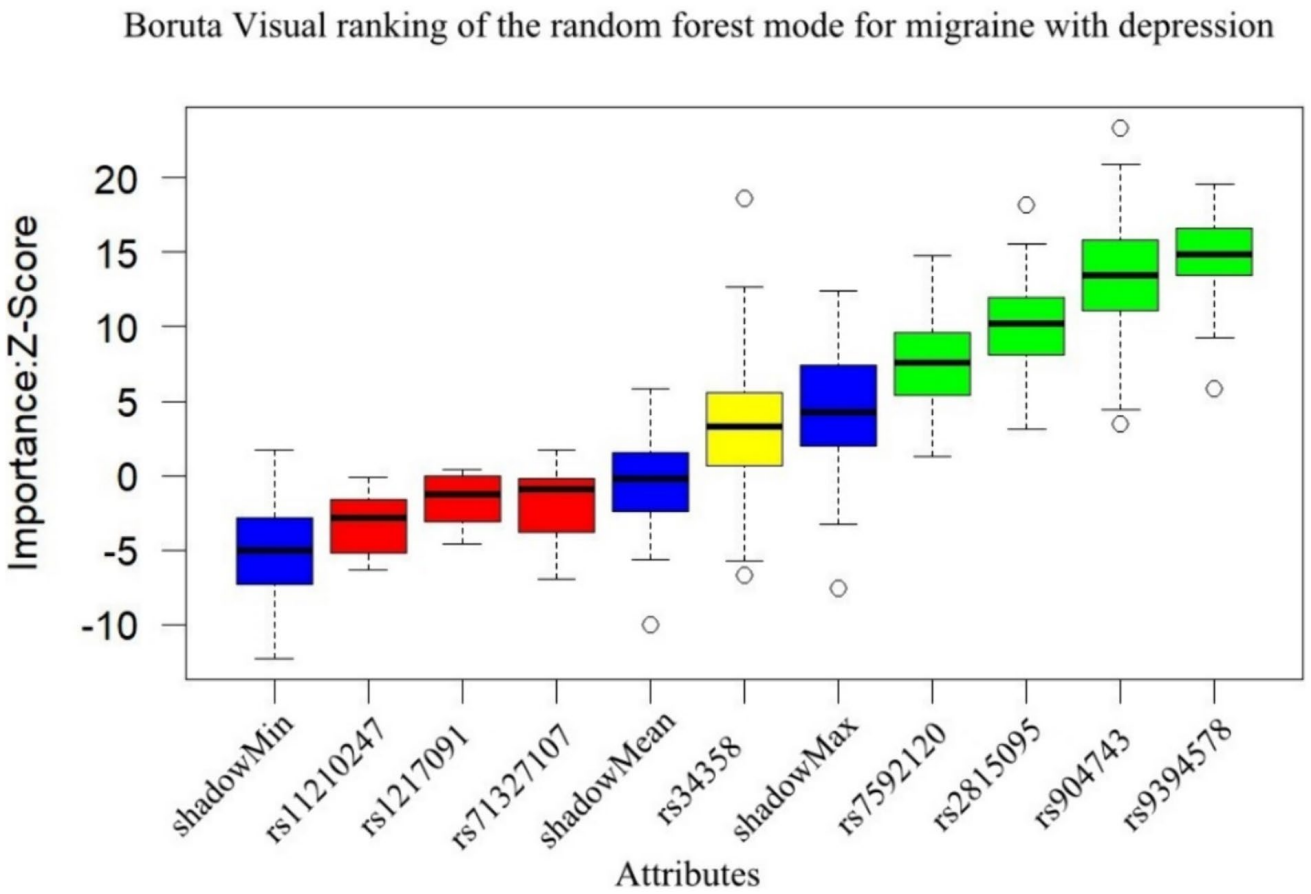
Results of Boruta algorithm for migraine with depression. Green is the important feature, yellow is the pending feature, and red is the rejected feature, and rs9394578, rs904743, rs2815095, rs7592120 were selected as important features for migraine with depression.

The subgroup analysis results show that the Boruta algorithm identified rs9394578, rs904743, rs2815095, and rs7592120 as significant features of migraine with depression (see Figure 2). According to the GLM logistic analysis, only rs904743 was a significant risk (OR = 1.64, 95% CI: 1.08–2.50, *p* = 0.021) after Bonferroni correction (see Table 1).

**Figure 3:**
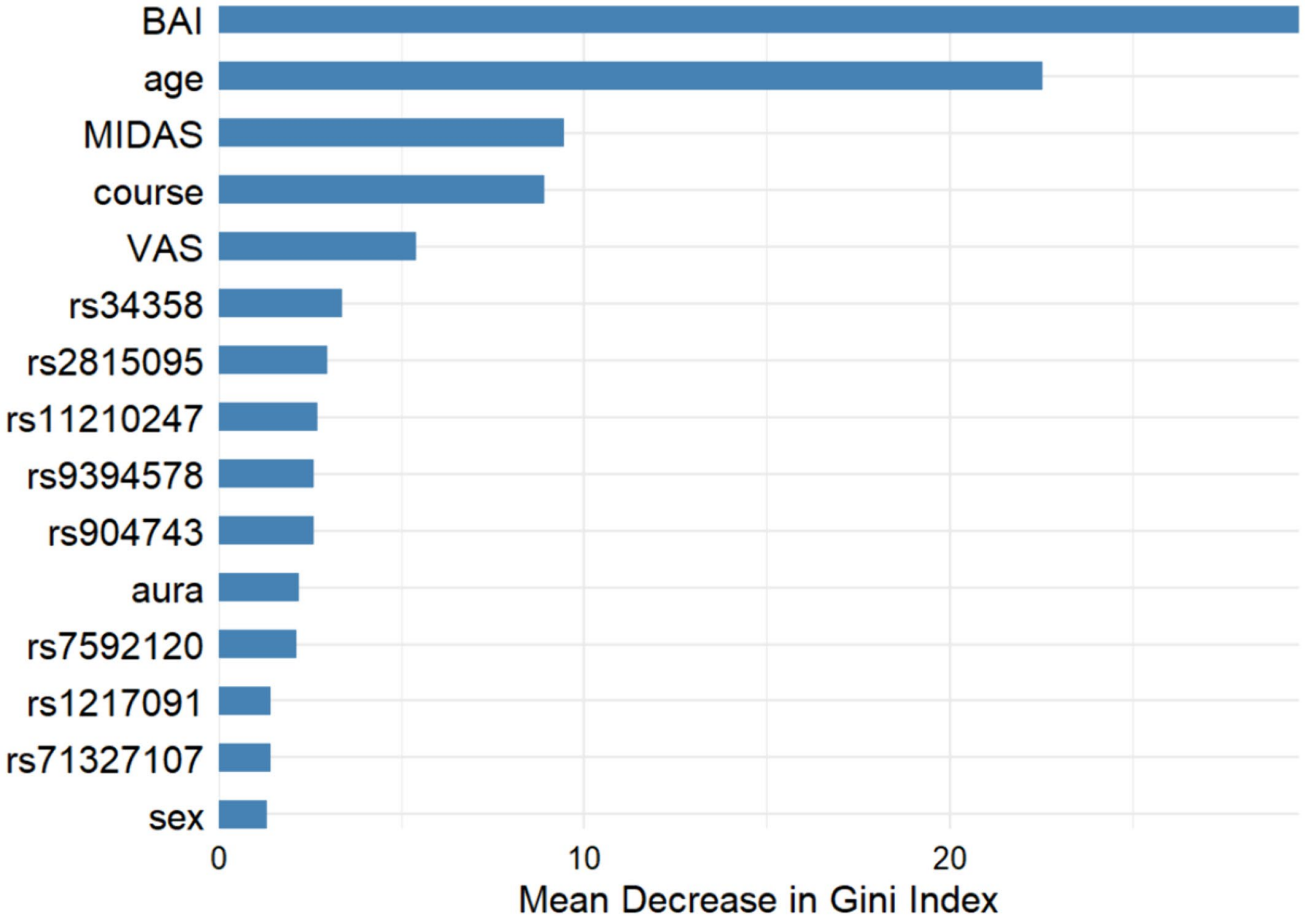
Machine learning results for migraine variables with and without depression. (1) Gini index: higher values indicate a greater role. (2) GLM logistic regression analysis suggested that the *p*-values for BAI, age, MIDAS was <0.001, 0.184, <0.001 respectively.

Figure 3 shows the comparison of clinical and genetic characteristics between migraine with and without depression, and it was found that age, BAI and MIDAS were important difference factors, but only BAI and MIDAS reached statistical difference (both *p* < 0.001). Other clinical and genetic characteristics were not statistically different between the two groups.

## Discussion

Our study confirmed that *ANKDD1B* rs904743 may increase susceptibility to migraine with depression in a Chinese population. This finding strengthens the reliability of previous GWAS studies (12). Other SNPs could not be replicated in this study, probably due to ethnic differences. Furthermore, the higher anxiety scores found in our study in migraine combined with depression, together with their higher MIDAS scores, can largely be explained by the fact that environmental stress and disease severity increase each other’s risk. The pathogenesis of co-morbidity between migraine and depressive disorders may involve structural and functional changes in the brain, abnormalities in neurotransmitter function, genetic factors, and environmental and stress factors, and these may be the direction of future treatment (18). Two epidemiological studies of large twins found a bidirectional association between migraine and depression, with 20% of the variability in depression and migraine attributable to shared genes (19, 20). In this study, *ANKDD1B* rs904743 was associated not only with depression combined with migraine, but also with migraine susceptibility, suggesting that *ANKDD1B* gene may be a common genetic basis for both disorders.

Previous studies have found several genes that may be associated with migraine combined with depression, including the promoter region of the 5-HT transporter (5HTTLPR) *SLC6A4* gene (21), the dopamine receptor gene *DRD2* and *DRD4* (22), and the GABAergic system of the *GABRQ* and *GABRA3*genes (23). These findings support the existence of common neurotransmitter abnormalities between the two diseases. Based on the results of previous GWAS studies in migraine, Peter et al. applied two machine learning algorithms and found that *REST*, *HPSE2* and *ADGRL2* may be the main candidates for the pathophysiology of migraine combined with depression (24). However, the study by Lannie et al. suggests that migraine with and without depression are genetically distinct disorders (25). In a study based on microRNA (miRNA) biomarkers, 11 of the 12 miRNA biomarkers associated with migraine were found to be associated with major depression (26). These findings suggest that the two diseases may share complex mechanisms and shed light on developing new therapies for their treatment (27).

The function of *ANKDD1B* is predicted to be involved in signal transduction, particularly calcium homeostasis (28). Clinical studies have found that *ANKDD1B* variants may be associated with ankylosing spondylitis and hypertension (29, 30). Using large-scale summary statistics, Guo et al. found a significant association between diastolic blood pressure and migraine susceptibility, as well as identified five loci (*ITGB5*, *SMG6*, *ADRA2B*, *ANKDD1B*, and *KIAA0040*) that are shared biological factors for both blood pressure and migraine (28). While another study suggested a possible association between *ANKDD1B* and other genes and migraine in people of European ancestry by affecting lipoprotein subfractions (31). In the GWAS study, *ANKDD1B* and *KCNK5* were associated with migraine and MDD etiology as key genes in neural-related signaling pathways and ion channel regulatory pathways (12). The study confirmed the association between the *ANKDD1B* gene and comorbid depression in Chinese migraine patients. However, no correlation was found for *KCNK5*, which may be related to the polygenic effect of migraine comorbid depression and ethnic differences. Another population-based study in China found that *ANKDD1B* rs34358 was associated with a decreased risk of migraine as a protein-truncating variant (32). This is consistent with our overall findings, but there are differences at specific genetic locus, so further validation in a larger Chinese population is needed.

In this study, we used Random Forest and Boruta algorithms to visually rank the importance of features related to migraine comorbid depression, which gave us a more intuitive understanding of its risk factors (33). However, there is not necessarily a dependency between these selected features and the disease, so we further performed GLM analysis based on feature importance, which makes our results more convincing. Nevertheless, the study did not find the *ANKDD1B* rs904743 polymorphism to be a significant risk factor for migraineurs with or without comorbid depression. This also reflects some limitations of this study: firstly, due to the lack of long-term follow-up of migraine patients with concomitant depression during the study period, the slightly higher percentage of mildly depressed population may have attenuated the influence of genetic factors. Secondly, considering the complexity of the pathogenesis of migraine combined with depression, the sample size of this study was not large enough, and further studies with more centers and samples are needed.

## Conclusion

In summary, our findings indicate that the *ANKDD1B* rs904743 variant identified in genome-wide association study was significantly associated with an increased susceptibility to migraine accompanied by depression in Chinese patients. In addition, SNPs associated with other populations were not replicated in the Chinese population, suggesting the need for larger and more innovative studies to understand the mechanisms of migraine and depression comorbidity at the genetic level.

## Data availability statement

The datasets presented in this study can be found in online repositories. The names of the repository/repositories and accession number(s) can be found in the article/Supplementary material.

## Ethics statement

The studies involving humans were approved by the project was approved by the Ethics Committee of the First Affiliated Hospital of Xiamen University (XMYY-2021KYSB009). The studies were conducted in accordance with the local legislation and institutional requirements. Written informed consent for participation in this study was provided by the participants’ legal guardians/next of kin.

## Author contributions

XA: Conceptualization, Funding acquisition, Investigation, Methodology, Project administration, Supervision, Visualization, Writing – original draft, Writing – review & editing. SZ: Data curation, Formal analysis, Investigation, Methodology, Resources, Software, Writing – review & editing, Writing – original draft. JF: Investigation, Methodology, Software, Validation, Visualization, Writing – review & editing. QL: Data curation, Methodology, Project administration, Software, Validation, Writing – review & editing. CY: Formal analysis, Methodology, Resources, Software, Supervision, Writing – review & editing. CJ: Formal analysis, Methodology, Project administration, Resources, Supervision, Writing – review & editing. YZ: Data curation, Formal analysis, Investigation, Methodology, Resources, Writing – review & editing. JaZ: Investigation, Methodology, Project administration, Resources, Validation, Writing – review & editing. JeZ: Investigation, Methodology, Project administration, Resources, Software, Writing – review & editing. CC: Investigation, Methodology, Software, Supervision, Validation, Writing – review & editing. HQ: Conceptualization, Investigation, Project administration, Resources, Supervision, Writing – review & editing. QM: Conceptualization, Data curation, Funding acquisition, Investigation, Methodology, Resources, Writing – review & editing. QL: Conceptualization, Funding acquisition, Methodology, Project administration, Resources, Supervision, Visualization, Writing – review & editing.

## Footnotes

^1^ https://www.r-project.org/
