# Supplementary material for: Identification of genetic susceptibility for Chinese migraine with depression using machine learning
Source: Front Neurol. 2024 Jul 31;15:1418529. doi: 10.3389/fneur.2024.1418529 (PMC11322385; doi:10.3389/fneur.2024.1418529)
Supplement: Supplementary file 1 [file Table_1.docx]

Supplemental Table 1 Comparison of clinical characteristics of study participants

|  | Migraine (266) | Control (223) | *P* |
| --- | --- | --- | --- |
| Gender |  | | |
| Male (%) | 30(11.3) | 32(14.3) | 0.309 |
| Female (%) | 236(88.7) | 191(85.7) |  |
| Age | 34.0±9.3 | 33.1±8.5 | 0.245 |
| Classification |  | | |
| Migraine with aura (%) | 36(13.5) | —— | —— |
| migraine without aura (%) | 230(86.5) | —— | —— |
| Comorbid depression | 108（40.6） |  |  |
