# Supplementary material for: Identification of genetic susceptibility for Chinese migraine with depression using machine learning
Source: Front Neurol. 2024 Jul 31;15:1418529. doi: 10.3389/fneur.2024.1418529 (PMC11322385; doi:10.3389/fneur.2024.1418529)
Supplement: Supplementary file 2 [file Table_2.docx]

**Supplementary Table 2. Genotype and Allele Frequencies of gene polymorphism in Chinese Migraine Case-Control Population**

| SNP | Group | Genotypes | | | *P* | Alleles | | *P* |
| --- | --- | --- | --- | --- | --- | --- | --- | --- |
| rs34358  (*ANKDD1B*) |  | GG | GA | AA |  | G | A |  |
|  | Migraine | 106 (39.9) | 123(46.2) | 37(13.9) | 0.868 | 335(63.0) | 197(37.0) | 0.603 |
|  | Control | 94(42.2) | 100(44.8) | 29(13.0) |  | 288(64.6) | 158(35.4) |  |
| rs904743  (*ANKDD1B*) |  | AA | AG | GG |  | A | G |  |
|  | Migraine | 186(69.9) | 63(23.7) | 17(6.4) | **0.016**^1^ | 435(81.8) | 97(18.2) | **0.027**^1^ |
|  | Control | 130(58.3) | 79(35.4) | 14(6.3) |  | 339(76.0) | 107(24.0) |  |
| rs9394578  (*KCNK5*) |  | CC | CA | AA |  | C | A |  |
|  | Migraine | 203(76.3) | 54(20.3) | 9(3.4) | 0.073 | 460(86.5) | 72(13.5) | 0.273 |
|  | Control | 174(78.1) | 48(21.5) | 1(0.4) |  | 396(88.8) | 50(11.2) |  |
| rs2815095  (*KCNK5*) |  | CC | CT | TT |  | C | T |  |
|  | Migraine | 201(75.5) | 59(2.2) | 6(2.3) | 0.258 | 461(86.7) | 71(13.3) | 0.637 |
|  | Control | 169(75.8) | 53(23.8) | 1(0.4) |  | 391(87.7) | 55(12.3) |  |
| rs1217091 |  | CC | CT | TT |  | C | T |  |
|  | Migraine | 246(92.5) | 20(7.5) | 0(0) | 0.458 | 512(96.2) | 20(3.8) | 0.466 |
|  | Control | 210(94.2) | 13(5.8) | 0(0) |  | 433(97.1) | 13(2.9) |  |
| rs7592120 |  | TT | TC | CC |  | T | C |  |
|  | Migraine | 197(74.1) | 66(24.8) | 3(1.1) | 0.108 | 460(86.5) | 72(13.5) | 0.713 |
|  | Control | 167(74.9) | 48(21.5) | 8(3.6) |  | 382(85.7) | 64(14.3) |  |
| rs11210247 |  | CC | CT | TT |  | C | T |  |
|  | Migraine | 151(56.8) | 105(39.5) | 10(3.7) | 0.213 | 407(76.5) | 125(23.5) | 0.111 |
|  | Control | 144(64.6) | 72(32.3) | 7(3.1) |  | 360(80.7) | 86(19.3) |  |
| rs71327107 |  | TT | TG | GG |  | T | G |  |
|  | Migraine | 227(85.3) | 38(14.3) | 1(0.4) | 0.369 | 492(92.5) | 40(7.5) | 0.155 |
|  | Control | 180(80.7) | 41(18.4) | 2(0.9) |  | 401(86.5) | 45(13.5) |  |

1. Migraine compared with control by A allele: OR= 1.415, 95% CI: 1.039~1.929, *P* = 0.027.
